# The effect of three different exercise training modalities on cognitive and physical function in a healthy older population

**DOI:** 10.1186/s11556-017-0183-5

**Published:** 2017-08-10

**Authors:** Carla Coetsee, Elmarie Terblanche

**Affiliations:** Department of Sport Science, Faculty of Education, Stellenbosch University, Private Bag X1, Matieland, 7601 South Africa

**Keywords:** Executive function, Cardiovascular fitness, Functional capacity, Stroop task, Older adults

## Abstract

**Background:**

Older adults are encouraged to participate in regular physical activity to counter the age-related declines in physical and cognitive health. Literature on the effect of different exercise training modalities (aerobic vs resistance) on these health-related outcomes is not only sparse, but results are inconsistent. In general, it is believed that exercise has a positive effect on executive cognitive function, possibly because of the physiological adaptations through increases in fitness. Indications are that high-intensity interval training is a potent stimulus to improve cardiovascular fitness, even in older adults; however, its effect on cognitive function has not been studied before.

Therefore, the purpose of this study was to compare the effects of resistance training, high-intensity aerobic interval training and moderate continuous aerobic training on the cognitive and physical functioning of healthy older adults.

**Methods:**

Sixty-seven inactive individuals (55 to 75 years) were randomly assigned to a resistance training (RT) group (*n* = 22), high-intensity aerobic interval training (HIIT) group (*n* = 13), moderate continuous aerobic training (MCT) group (*n* = 13) and a control (CON) group (*n* = 19) for a period of 16 weeks. Cognitive function was assessed with a Stroop task and physical function with the Timed-Up-and-Go (TUG) and submaximal Bruce treadmill tests.

**Results:**

No significant GROUP x TIME interaction was found for Stroop reaction time (*P* > .05). The HIIT group showed the greatest practical significant improvement in reaction time on the information processing task, i.e. Stroop Neutral (ES = 1.11). MCT group participants had very large practical significant improvements in reaction time on the executive cognitive tasks, i.e. Stroop Incongruent and Interference (ES = 1.28 and 1.31, respectively). The HIIT group showed the largest practically significant increase in measures of physical function, i.e. walking endurance (ES = 0.91) and functional mobility (ES = 0.36).

**Conclusions:**

MCT and RT proved to be superior to HIIT for the enhancement of older individuals’ executive cognitive function; whereas HIIT were most beneficial for improvement in information processing speed. HIIT also induced the largest gains in physical function.

## Background

The inevitable effects of ageing on the physical and mental health of humans can somewhat be offset by regular participation in physical exercise during adulthood [1]. Although an acute bout of exercise positively affects executive cognitive performance, regular exercise during midlife has a protective effect against cognitive decline in the later adult years [2].

Etnier & Chang [3] described executive function as “a higher order cognitive ability that controls basic, underlying cognitive functions for purposeful, goal-directed behaviour and that has been associated with frontal lobe activity.” Executive function refers to the domains of cognitive function that involve executive control, including planning, scheduling, working memory, interference control and task coordination [4]. Conceptually, executive function is considered critical for performance in novel situations or when an individual is required to inhibit a previously learned response [3].

McAuley et al. [5] asserted that a decline in executive cognitive control is associated with the normal ageing process. This proposed decline has been associated with changes (e.g. volumetric) in certain brain areas, especially the frontal lobes. Additionally, Royall et al. [6] demonstrated in a three-year cohort study that the regression in executive cognitive control is independently associated with longitudinal declines in functional status.

Tests designed to assess executive function usually represent external tasks that are unfamiliar or uncommon and requires an individual to apply certain intellectual abilities (e.g. planning) in order to solve the task/problem [7]. The Stroop task [8] is one of the most frequently used executive function measures. A number of modifications of this test (i.e. card-based and computerized) have been used in research and clinical settings. The Stroop task, consisting of conditions of increasing difficulty, is used to assess a number of executive function components, including selective attention, the ability to shift response/perceptual sets and the ability to inhibit habitual responses [9].

Recently, there has been growing interest in the promotion of physical activity to improve cognitive function and there is mounting evidence that exercise can positively influence and preserve this construct. Furthermore, it was suggested that the greatest effects are observed for higher level cognitive functions, such as executive function, with fewer effects on lower level functions [10]. Up till now the majority of longitudinal studies focussed on the effect of a single exercise training modality on executive cognitive function, i.e. aerobic exercise [11–13] or resistance exercise [14–17]. Studies comparing the effects of aerobic and resistance training on cognitive function are in the minority. Nevertheless, from what is known so far, it would seem that aerobic training yields the best results [18, 19].

The majority of longitudinal resistance training studies report improvements in executive cognitive processes [14–17, 20, 21]. It was also suggested that aerobic training improves performance on tasks which demand greater executive control processes, a phenomenon known as the “selective improvement” hypothesis [18, 22].

Aerobic training interventions generally have a more profound impact on cardiovascular fitness compared to resistance training [12, 19]. However, high-intensity aerobic interval training (HIIT) has been shown to induce larger increases in maximal aerobic capacity (VO_2max_) compared to aerobic training at a constant intensity [23, 24]. Consequently, as cardiovascular fitness has been proposed as a potential mediating factor in the enhancement of cognitive performance [13], the question regarding the effect of HIIT on cognition is a matter of interest.

Therefore, the present study aimed to determine if different exercise training modalities (resistance training, high-intensity aerobic interval training and moderate continuous aerobic training) have similar effects on the cognitive performance of older individuals, as assessed by a Stroop task. Furthermore, the effects of the different training interventions on measures of physical function, i.e. walking endurance and functional mobility, were investigated. The authors’ primary hypothesis was that high intensity interval aerobic training will have superior effects on the physical and cognitive function of older adults compared to resistance training and moderate continuous aerobic training. The secondary hypothesis was that the exercise training groups will show greater improvements on the executive function tasks compared to the information processing speed task.

## Methods

### Participants

Inactive men and women between 55 and 75 years old who volunteered for this intervention study underwent a screening procedure to identify those who met the inclusion criteria. All testing procedures were done at the Sport Science Department of Stellenbosch University, South Africa. Individuals were included if they: (a) had a body mass index (BMI) of less than 35 kg/m^2^; and (b) had not been participating in at least 30 min of moderate intensity physical activity (64%–76% of maximal heart rate) on at least 3 days of the week for the previous 3 months. Participants were excluded if they: (a) had one or more signs/symptoms of, or diagnosed cardiovascular, pulmonary and/or metabolic diseases; (b) experienced orthopaedic or musculoskeletal problems that could affect their exercise ability; (c) achieved a Montreal Cognitive Assessment (MoCA) score of less than 26 out of 30; and (d) if they were on any medications that may affect cognitive function or heart rate. The study proposal was approved by the Ethics Committee of Stellenbosch University (HS891/2013).

Of the 82 volunteers who were screened, 72 met the inclusion criteria and were randomly assigned to either a resistance training (RT) group, high-intensity aerobic interval training (HIIT) group, moderate continuous aerobic training (MCT) group or a non-exercise control (CON) group. All participants were informed of the purpose of the study and gave written consent to participate. Two participants dropped out of the RT group, while three did not want to participate because they were included in the CON group. Two participants dropped out of the HIIT group as a result of injury (unrelated to the study). However, their data were included in the data set until the point of departure. Thus, 67 men and women (mean age 62.7 ± 5.7 years; BMI 26.4 ± 4.0 kg/m^2^) started the intervention, with 22 participants in the RT group (men/women ratio: 7/15), 13 in the HIIT group (men/women ratio: 3/10), 13 in the MCT group (men/women ratio: 3/10) and 19 in the CON group (men/women ratio: 8/11) (Table 1).

**Table 1:** Baseline characteristics of the participants (mean ± SD)

Variable	HIIT group	MCT group	RT group	CON group
n	13	13	22	19
Gender ratio (M:W)	3:10	3:10	7:15	8:11
Age (years)	64.5 ± 6.3	61.6 ± 5.8	62.4 ± 5.1	62.5 ± 5.6
Height (cm)	166 ± 8.9	163.5 ± 8.6	167.8 ± 7.8	168.7 ± 7.9
Body mass (kg)	73.8 ± 13.7	71.0 ± 14.4	73.3 ± 15.5	76.8 ± 13.7
BMI (kg·mˉ^2^)	26.6 ± 4.0	26.5 ± 4.2	25.8 ± 4.0	26.9 ± 3.7
VO_2peak_ (ml·kg ˉ^1^·min ˉ^1^)	17.3 ± 3.2	19.2 ± 6.0	19.4 ± 3.5	20.1 ± 4.0
MoCA score	27.9 ± 1.5	27.6 ± 1.3	27.5 ± 1.3	28.2 ± 1.6

No statistically significant differences in the physical characteristics of the groups at BL (*P* > .05)

*CON* control, *RT* resistance training, *HIIT* high-intensity aerobic interval training, *MCT* moderate continuous aerobic training, *BL* baseline, *BMI* body mass index, *VO*_*2peak*_ peak oxygen uptake, *MoCA* Montreal Cognitive Assessment

### Testing protocol

Cognitive performance and physical function were measured as primary outcome variables and were assessed at baseline (BL) and at the end of the intervention period (week 16). Participants were asked to refrain from smoking and exercise for at least 4 and 12 h before the tests, respectively, as well as to maintain their current lifestyle and not make any changes to their level of physical activity and diet.

A resting ECG, waist-to-hip ratio, BMI and the MoCA [25] were administered during the first visit as screening tests. During the second visit (BL-testing) cognitive performance was assessed with a computerized Stroop task, which consisted of two blocks of increasing difficulty. Each block consisted of 24 trials during which the participant had to respond to a pre-determined command given at the beginning of the block. The first trial of each block served as familiarization to the specific condition. The stimulus was presented on the centre of a laptop screen with the two responses situated at the bottom left and right of the screen. Participants were instructed to use only the left and right arrow keys when responding to the stimulus. They were also given a choice with regards to the language (English or Afrikaans) in which they wanted to complete the task.

Initially, participants had to identify the colour of a rectangle with the choices written in black ink (Stroop Neutral). The next condition (Stroop Incongruent) required participants to identify the ink colour of a word written in incongruent coloured inks and thus disregard the semantic meaning of the word (e.g. the word “blue” printed in red ink), with the choices written in black ink. The degree of task difficulty increased in the latter condition, testing the participant’s ability to inhibit a prepotent/automated response. Participants’ reaction time and accuracy were measured for each trial. The colour subtask evaluated speed of information processing, whereas the incongruent colour-word subtask assessed components of executive function. Stroop Interference was calculated by subtracting the reaction time for the Neutral condition from the reaction time for the Incongruent condition and was also used as a measure of executive cognitive function.

The Timed-Up-and-Go (TUG) test was administered to assess functional mobility [26]. The participant was instructed to sit on a standard chair. On the command “Go”, he/she stood up from the chair, walked three meters forward, turned and walked back to the chair. Timing started when the command was given and stopped when the individual was again sitting in the chair. Each participant performed three trials and the fastest time was noted as the final result.

The participant’s walking endurance was assessed on the h/p/cosmos Saturn treadmill (Nussdorf-Traunstein, Germany) using the Bruce protocol [27]. Heart rate was recorded with a Suunto memory belt (Suunto Oy 11/2007, Finland). The test started at an incline of 10 degrees and a speed of 2.7 km/h. The incline and speed were increased incrementally every 3 min until the target heart rate (THR) of 75% of the age-predicted maximal (220-age) was reached. The participant’s rating of perceived exertion (RPE) was recorded at the end of each stage and when the THR was reached. Participants then actively cooled down for 5 min at 2.7 km/h at zero incline. Peak oxygen uptake (VO_2peak_) was estimated from participants’ walking endurance time (minutes) using the formula of Foster et al. [28]: VO_2peak_ (ml·kg ˉ^1^·min ˉ^1^) = 14.760–1.379 (time) + 0.451 (time^2^) – 0.012 (time^3^).

### Training programmes

The intervention was conducted over a period of 16 weeks and participants completed three training sessions per week. Participants in the RT group performed upper and lower body resistance exercises using machines and free weights. Three sets of 10 repetitions were performed at 50%, 75% and 100% of the individual’s 10 repetition maximum (RM). After 8 weeks the load for each set was increased to 75%, 85% and 100% of the individual’s 10RM. The MCT group performed continuous walking on a treadmill at 70–75% of maximal heart rate (HRmax) for 47 min. The HIIT group performed four intervals of 4 min treadmill walking at 90–95% HRmax, interspersed by 3 min active recovery periods at 70% HRmax. The speed and inclination of the treadmill were continuously adjusted to ensure that participants trained at the correct intensity. The MCT and HIIT training sessions were isocaloric according to a study by Wisløff et al. [29]. The duration of each RT and HIIT session was approximately 30 min, excluding the warm-up and cool down.

### Statistical analysis

Statistical analysis was performed using STATISTICA 12. Probability plots were inspected to assess the normality of the data and check for outliers, and were found to fit the assumptions of a Gaussian distribution.

Mixed model repeated measures ANOVA was used to analyse the data. Group and time was entered as fixed effects. Participant, nested in group, was entered as random effect. Fisher’s least significant difference post-hoc tests were included to determine differences in treatment effects between groups. A *P* value of < .05 was considered statistically significant. Cohen’s effect sizes (ES) were calculated to quantify the magnitude of differences in outcome variables between the participant groups. These statistics were included as they are, unlike *P* values, independent of sample size and provide information on the size of the observed effect. Cohen’s thresholds of 0.2, 0.5, 0.8 and 1.2 were interpreted as small, moderate, large and very large effects, respectively [30]. Data are reported as means ± SD.

## Results

### Cognitive function

All participants achieved a MoCA score of more than 25 out of 30, indicating normal cognitive functioning. There were no statistically significant differences in the baseline (BL) physical and physiological characteristics of the different study groups (*P* > .05) (Table 1).

### Reaction time during the Stroop neutral task

Table 2 depicts the changes in Stroop performance (reaction time) after the 16-week intervention period. There were no statistically significant differences at baseline in reaction time between the groups for any of the Stroop subtasks (*P* > .05). The HIIT and RT groups showed showed large practically and statistically significant improvements in reaction time from pre- to post-test on the Stroop Neutral subtask (from 28.42 ± 5.76 s to 23.16 ± 2.85 s vs 30.03 ± 5.22 s to 25.51 ± 3.65 s; ES = 1.11 and 1.00, respectively; *P* < .05), while the MCT and CON groups improved moderately, but not statistically significantly (from 25.42 ± 2.33 s to 23.81 ± 2.34 s vs 28.85 ± 5.02 s to 25.67 ± 3.90 s; ES = 0.69 and 0.70, respectively; *P* > .05). Overall, there was a 18.51%, 15.05%, 6.33% improvement in lower level cognitive function following the HIIT, RT and MCT intervention, respectively, while the performance of the CON group improved by 11.02%. The GROUP x TIME interaction was not statistically significant, however, a significant TIME effect was evident at post-test (*P* < .001).

**Table 2:** Comparison of changes in Stroop reaction time (s) for all the groups before and after the 16-week intervention period (mean ± SD)

	HIIT group (*n* = 11)			MCT group (*n* = 13)			RT group (*n* = 22)			CON group (*n* = 19)		
Stroop task	Reaction time (s)	% change	Effect size	Reaction time (s)	% change	Effect size	Reaction time (s)	% change	Effect size	Reaction time (s)	% change	Effect size
Neutral												
Before	28.42 ± 5.76			25.42 ± 2.33			30.03 ± 5.22			28.85 ± 5.02		
After	23.16 ± 2.85	−18.51	1.11*	23.81 ± 2.34	−6.33	0.69	25.51 ± 3.65	−15.05	1.00*	25.67 ± 3.90	−11.02	0.70
Incongruent												
Before	41.62 ± 11.18			45.98 ± 10.71			53.60 ± 17.48			49.86 ± 16.75		
After	34.21 ± 6.37	−17.80	0.79	35.0 ± 5.72	−23.88	1.28*	38.94 ± 6.82	−27.35	1.12*	37.48 ± 8.30	−24.83	0.94*
Interference												
Before	13.96 ± 7.79			20.56 ± 9.26			23.85 ± 14.50			21.01 ± 13.81		
After	11.05 ± 4.31	−20.85	0.45	11.19 ± 3.99	−45.57	1.31*	12.30 ± 4.68	−48.43	1.07*	10.93 ± 5.42	−47.98	0.95*

*HIIT* high-intensity aerobic interval training, *MCT* moderate continuous aerobic training, *RT* resistance training, *CON* control

^*^Significantly different from pre-test (*P* < 0.05)

### Reaction time during the Stroop incongruent task

Table 2 shows that there were large practically significant within-group improvements in reaction time during the Incongruent Stroop subtask for all the groups after the 16 weeks, with the MCT group (from 45.98 ± 10.71 s to 35.0 ± 5.72 s; ES = 1.28; *P* < .05) and RT group (from 53.60 ± 17.48 s to 38.94 ± 6.82 s; ES = 1.12; *P* < .05) performing practically significantly better compared to the HIIT (from 41.62 ± 11.18 s to 34.21 ± 6.37 s; ES = 0.79; *P* > .05) and CON groups (from 49.86 ± 16.75 s to 37.48 ± 8.30 s; ES = 0.94; *P* < .05). Overall, there was a 23.88%, 27.35%, 17.80% improvement in higher level (executive) cognitive function following the MCT, RT and HIIT intervention, while the CON group’s performance improved by 24.83%. The GROUP x TIME interaction was not statistically significant, however, a significant TIME effect was evident at post-test (*P* < .001).

### Reaction time during the Stroop interference task

An improvement in reaction time was evident in the Stroop Interference subtask for all the groups from pre- to post-test, following the same trend that was observed for the Incongruent Stroop subtask (Table 2). The MCT and RT groups performed practically and statistically significantly better (from 20.56 ± 9.26 s to 11.19 ± 3.99 s vs 23.85 ± 14.50 s to 12.30 ± 4.68 s; ES = 1.31 and 1.07, respectively; *P* < .05) compared to the HIIT and CON groups (from 13.96 ± 7.79 s to 11.05 ± 4.31 s; ES = 0.45; *P* > .05 vs 21.01 ± 13.81 s to 10.93 ± 5.42 s; ES = 0.95; *P* < .05 respectively). Overall, there was a 45.57%, 48.43%, 20.85% improvement in higher level (executive) cognitive function following the MCT, RT and HIIT intervention, while the performance of the CON group improved by 47.98%. The GROUP x TIME interaction was not statistically significant, however, a significant TIME effect was evident at post-test (*P* < .001).

### Task accuracy for the Stroop neutral and incongruent tasks

Table 3 shows the accuracy (% correct) of the participants during the Neutral and Incongruent Stroop tasks before and after the 16-week intervention period. There were no statistically significant main or interaction effects for task accuracy on the Neutral task (*P* > .05). All the participants achieved more than 98% accuracy, with only the RT group showing an improvement of moderate practical significance from pre- to post-test (ES = 0.61; *P* > .05).

**Table 3:** Comparison of changes in task accuracy (% correct) on the Stroop conditions for all the groups before and after the 16-week intervention period (mean ± SD)

Stroop task	HIIT group (*n* = 11)			MCT group (*n* = 13)			RT group (*n* = 22)			CON group (*n* = 19)		
	Accuracy (%)	% change	Effect size	Accuracy (%)	% change	Effect size	Accuracy (%)	% change	Effect size	Accuracy (%)	% change	Effect size
Neutral												
Before	98.91 ± 1.88			98.55 ± 2.71			98.55 ± 2.05			99.28 ± 1.62		
After	98.81 ± 1.94	−0.10	−0.05	99.0 ± 1.83	0.46	0.19	99.59 ± 1.28	1.06	0.61	99.52 ± 1.37	0.24	0.16
Incongruent												
Before	93.31 ± 6.95			97.10 ± 3.24			94.0 ± 6.77			98.55 ± 3.24		
After	95.22 ± 4.94	2.05	0.31	96.74 ± 2.59	−0.37	−0.12	96.27 ± 4.89	2.41	0.39	96.80 ± 3.10	−1.78	−0.55

*HIIT* high-intensity aerobic interval training, *MCT* moderate continuous aerobic training, *RT* resistance training, *CON* control

There were also no statistically significant main or interaction effects for task accuracy on the Incongruent task (*P* > .05). All the participants’ accuracy were more than 90%, with the three exercise training groups showing small changes from pre-test to post-test (ES < 0.4; *P* > .05). The CON group, however, exhibited a decrease in task accuracy of moderate practical significance, scoring a higher error rate on the Incongruent Stroop task after the 16-week period (98.55 ± 3.24% vs 96.80 ± 3.10%; ES = −0.55; *P* > .05).

### Walking endurance

Table 4 depicts the effect of the training programmes on the results of the Bruce treadmill test. There was a large practically and statistically significant improvement in walking endurance in the HIIT group after 16 weeks (1.42 ± 1.32 min; ES = 0.91; *P* < .05), followed by a near moderate practical significant, but statistically non-significant improvement in the RT group (0.72 ± 0.86 min; ES = 0.48; *P* > .05) and a trivial increase in the MCT group (0.64 ± 1.03 min; ES = 0.16; *P* > .05). There was no meaningful change in the walking time of the CON group (−0.00 ± 0.69 min; ES = 0.00; *P* > .05). There was a statistically significant GROUP x TIME interaction for submaximal endurance capacity (*P* < .05).

**Table 4:** Comparison of changes in physical function for all the groups before and after the 16-week intervention period (mean ± SD)

Outcome measure	HIIT group (*n* = 11)	% change	Effect size	MCT group (*n* = 13)	% change	Effect size	RT group (*n* = 22)	% change	Effect size	CON group (*n* = 19)	% change	Effect size
Bruce test (min)												
Before	4.40 ± 1.74			4.96 ± 2.51			5.46 ± 1.57			5.76 ± 1.59		
After	5.91 ± 1.54	34.24	0.91*	5.30 ± 1.52	6.77	0.16	6.17 ± 1.40	13.16	0.48	5.75 ± 1.56	−0.05	0.00
TUG test (s)												
Before	5.58 ± 0.73			5.60 ± 0.67			5.36 ± 0.92			5.53 ± 1.10		
After	5.32 ± 0.7	−4.60	0.36	5.41 ± 0.81	−3.54	0.27	5.13 ± 0.75	−4.28	0.27	5.67 ± 0.81	2.35	0.13

*HIIT* high-intensity aerobic interval training, *MCT* moderate continuous aerobic training, *RT* resistance training, *CON* control

*Significantly different from pre-test (*P* < 0.05)

### Functional mobility (TUG)

A statistically significant GROUP x TIME interaction for TUG performance (*P* < .05) was noted. There was a small practically significant increase in TUG performance in the HIIT group after 16 weeks of training (−0.30 ± 0.37 s; ES = 0.36; *P* > .05), compared to the RT (−0.23 ± 0.55 s; ES = 0.27; *P* > .05) and MCT (−0.20 ± 0.26 s; ES = 0.27; *P* > .05) groups (Table 4). Participants in the CON group performed slightly worse after the intervention period (0.24 ± 0.54 s; ES = 0.13; *P* > .05).

## Discussion

To the best of our knowledge, this is the first attempt to compare the effects of high-intensity aerobic interval training to traditional (continuous) aerobic training, as well as resistance training on cognitive function in older adults. Moderate continuous aerobic training (MCT) proved to be most beneficial for the enhancement of executive cognitive function, a higher level cognitive process; whereas high-intensity aerobic interval training (HIIT) had the greatest positive effect on information processing speed, a lower level cognitive process. Resistance training (RT) at a moderate intensity was more beneficial for gains in information processing speed compared to MCT, and executive cognitive function compared to HIIT. As hypothesized, the greatest improvement in physical function, i.e. walking endurance and functional mobility, was induced by HIIT. However, HIIT was the only study group that did not show a statistically significant improvement in performance on the executive function tasks, a finding contradictory to the authors’ hypothesis.

Accuracy on the two Stroop tasks did not change significantly after the 16-week period in either of the study groups. A moderate decrease in accuracy on the Incongruent task was, however, observed in the control (CON) group after the intervention period. It is therefore suggested that the CON group’s improvement in reaction time on the Incongruent Stroop task could be due to a speed-accuracy trade off.

The cognitive function results of the present study, specifically the changes exhibited by the MCT group, provide support for the “selective improvement” hypothesis, as proposed by Kramer et al. [18]. These authors also observed a selective effect of aerobic exercise on cognitive function. Thus, other performance measures that were not linked to executive function remained unaffected. Furthermore, our results are in agreement with previous intervention studies which proposed that different types of exercise interventions have unique effects on cognition [31]. In addition, our results extend the existing literature by adding novel findings with regards to the effect of HIIT on cognitive function.

Traditional moderate-intensity aerobic training showed the greatest benefit on tasks assessing executive function, with no significant improvements in information processing speed. The findings of Predovan et al. [13] also provide a degree of support for the hypothesis that traditional aerobic exercise training has a selective effect on cognition. After a 12-week intervention, the training group improved their performance in the inhibition/switching (i.e. set shifting) task (Stroop Interference), which was considered to recruit the highest level of executive functioning (multiple executive processes), but no training effect was found for the naming (Neutral) or inhibition (Incongruent) conditions of the Stroop task. They therefore suggested that aerobic dancing selectively improves tasks assessing switching and not necessarily tasks requiring inhibitory processes. The researchers proposed that differences in the type of aerobic exercises performed (i.e. aerobic dancing, walking, running etc.) could explain the inconsistent findings across studies [13].

In contrast to the findings of the present study, some investigators observed a beneficial effect of aerobic training at a constant, moderate intensity on tasks assessing information processing speed (lower level cognitive function) in addition to executive function. A meta-analytic review by Smith et al. [32] reported modest improvements in older adults’ lower and higher level neurocognitive functions after participation in aerobic training interventions that lasted between 6 weeks and 18 months. These improvements were observed for attention and processing speed, executive function and memory; whereas working memory did not benefit from aerobic training [32]. Additionally, Dustman et al. [12] found that 4 months of aerobic training resulted in significantly better scores on simple and complex cognitive tasks compared to strength and flexibility training.

In the present study, aerobic interval training at alternating periods of high and low intensities significantly improved performance on a task assessing information processing speed, whereas no improvement was found on the executive function tasks. Thus, both the HIIT and MCT interventions induced selective improvements in cognitive function, albeit different cognitive domains were affected.

In a more recent study, conducted by Iuliano et al. [31], 12 weeks of cardiovascular training at higher intensities significantly improved attention and abstract reasoning; whereas no improvement was observed in older adults’ executive function. The dissimilarities in the study outcomes could be attributed to the differences in training stimuli (i.e. shorter duration training sessions at a higher intensity in the former study vs longer duration training sessions at a moderate intensity in the present study).

In the present study RT at a moderate intensity was more beneficial for gains in information processing speed compared to MCT, and executive cognitive function compared to HIIT. These findings do not support the notion that RT has a selective effect on cognition, as positive results were obtained across all domains (higher and lower level) of cognitive function measured. The majority of previous intervention studies reported a selective improvement of RT on cognitive function, with executive control tasks showing the greatest improvement [14–17, 20, 21]. Additionally, positive associations have been demonstrated between greater lower extremity strength and better executive function [33], as well as muscle-strengthening activities and executive function [34]. Researchers observed an improvement in the Stroop Incongruent task in older adults after 4 weeks of RT, however, the same effect was not found on a task assessing information processing speed [14], amounting to a selective effect on cognitive function. This finding is contradictory to the positive effect of RT found for both executive function and information processing speed in the present study. One could argue that the study duration of 4 weeks chosen by Anderson-Hanley et al. [14] was too short to induce noteworthy improvements in information processing speed, but that longer duration RT can have beneficial effects. Liu-Ambrose et al. [16] reported an improvement in the Stroop Interference task after 12 months of RT, compared to balance and tone exercises in community-dwelling older women. Smiley-Oyen et al. [19] found an improvement in the Stroop Incongruent task after 10 months of aerobic training in older men and women, but in contrast to the results of the present study, no effect was observed in the strength-and-flexibility training group. This group performed exercises with resistance bands, free weights and stability balls. It could be argued that the intensity of the strength exercises was too low to provide a sufficient stimulus for cognitive improvements. Furthermore, their results could only partly corroborate the “selective improvement” hypothesis, as positive results were not obtained across all the tasks assessing executive function. The researchers neglected to include a no-exercise control group, leading one to question whether the results can be solely attributed to the exercise.

As hypothesized, the HIIT group experienced the largest improvement in walking endurance compared to the other training groups. This is not an uncommon finding in the literature [23, 24]. Helgerud et al. [23] found that healthy, trained men who exercised at higher intensities (90–95% HR_max_) exhibited the biggest gains in aerobic capacity, whereas those exercising at lower intensities (70 and 85% HR_max_) did not improve their VO_2max_. This finding, as well as the results of the current study, supports the notion that cardiovascular adaptations to training (i.e. improvements in VO_2max_) are intensity dependent [35].

A surprising finding was that the MCT group did not experience an improvement in walking endurance, while the RT group actually showed a greater practically significant improvement in walking endurance compared to the MCT group. It is generally accepted that aerobic training leads to larger increases in aerobic capacity compared to resistance training [12, 19]. However, while some investigators observed no effect of RT on VO_2max_ [20], others reported a beneficial effect on VO_2max_ and walking endurance [36–38].

The results exhibited by the MCT group are also inconsistent with the findings reported by Dustman et al. [12], where a pronounced increase in VO_2max_ and Stroop Interference was observed after 16 weeks of aerobic training. There may be two possible explanations for these conflicting findings: (a) differences in computing the Stroop Interference effect and (b) dissimilarities in the exercise protocols. Dustman et al. [12] subtracted the reaction time for the Stroop Word task from the Incongruent task. However, the Interference score in the present study was calculated as the difference in reaction time between the Neutral and Incongruent tasks, similar to a more recent intervention study [16]. Furthermore, the intensity reported in Dustman’s study was higher than the exercise intensity used in the present study. It could thus be argued that our training stimulus was too low for the MCT group to induce significant improvements in aerobic capacity.

Mechanisms such as angiogenesis, synaptogenesis and neurogenesis have been proposed as possible mediating factors in the exercise-cognition relationship. Increases in biological mediators, including brain-derived neurotrophic factor (BDNF) and insulin-like growth factor 1 (IGF-1), have also been linked to exercise training [15, 39]. However, it has been suggested that the upregulation of these biological mediators are not dependent on aerobic fitness [19] and that neurocognitive networks are differentially influenced by the exercise training mode [40]. Aerobic training is linked to elevated levels of BDNF [39], while resistance training produces increased levels of IGF-1 [15]. These dissimilarities could possibly serve as an explanation for the differential effects of exercise training mode on cognitive function observed in the present study.

Positive effects of aerobic training on cognitive function and cerebral oxygenation during cortical activation have been recently reported in the literature [41]. We previously demonstrated that changes in cerebral oxygenation are differentially influenced by the exercise training mode [42] and we therefore suggest that the MCT group, irrespective of their minimal changes in aerobic fitness, experienced the most profound structural and functional neural adaptations which enabled them to perform practically significantly better, i.e. more efficiently, on the executive function tasks compared to the other study groups.

## Conclusions

MCT and RT proved to be superior to HIIT for the enhancement of older individuals’ executive cognitive function; whereas HIIT were most beneficial for the improvement in information processing speed. HIIT also induced the largest gains in physical function, i.e. walking endurance and functional mobility.

### Implications for future research

The findings of the present study highlight the importance of longer duration exercise training sessions of a moderate intensity for gains in executive cognitive function. Future studies are needed to replicate our findings and determine the effects of high-intensity interval training on other domains of cognitive function. The link between increased aerobic fitness and gains in cognitive function also needs further investigation, while the role of oxygenation may shed light on a potential underlying mechanism.

This is the first study to examine the effect of HIIT on older individuals’ cognitive function. Our findings highlight the importance of this mode of training, in addition to traditional aerobic and resistance training, for the promotion of physical function in the older population. Future studies should investigate the long-term effects of HIIT on older individuals’ health and physical function. Interventions combining interval training with traditional training modes will be helpful to determine the most beneficial exercise prescription for healthy, older adults.

### Study limitations

The small sample size of each study group is a limitation to the present study. Furthermore, it cannot be excluded that there may be a learning effect when Stroop tasks are repeated. Even though the sequence of trials in each Stroop subtask was randomized for every individual at pre- and post-test, all the groups (including the CON group) showed an improvement in reaction time on the simple and complex cognitive tasks after the 16-week intervention period. The HIIT and MCT programmes were designed to be isocaloric and thus adds strength to the present study. A limiting factor, however, is that it could not be quantified whether the RT programme was isocaloric to the two aerobic training programmes. We can therefore not exclude these dissimilarities as a possible explanation for the differences observed in the study outcomes.
